# Editorial: Management of patients with dialysis dependent chronic kidney disease (DD-CKD)

**DOI:** 10.3389/fmed.2026.2009687

**Published:** 2026-10-13

**Authors:** Guido Gembillo, Xin Hu, Daqing Hong, Junhua Li, Prem Prakash Kushwaha

**Affiliations:** 1 Unit of Nephrology and Dialysis, AOU “G. Martino”, University of Messina, Messina, Italy; 2 National Center for Child Health and Development (NCCHD), Tokyo, Japan; 3 Sichuan Academy of Medical Sciences and Sichuan Provincial People’s Hospital, Chengdu, China; 4 Huazhong University of Science and Technology, Wuhan, China; 5 School of Medicine, Case Western Reserve University, Cleveland, OH, United States

**Keywords:** acute kidney injury (AKI), chronic kidney disease, dialysis complications, hemodialysis, kidney replacement therapy (KRT), peritoneal dialysis

Chronic kidney disease affects a substantial and growing share of the adult population worldwide, and kidney failure requiring kidney replacement therapy for survival affects approximately 0.2% of the global population (1). Patients maintained on haemodialysis or peritoneal dialysis carry cardiovascular disease, anaemia, mineral and bone disorder, infection, malnutrition and symptom load at the same time, and their management is guided by a limited set of laboratory and kinetic measures. How closely those measures track the outcomes patients experience is among the questions the contributions gathered here address. This Research Topic assembles eighteen contributions comprising original cohort and registry analyses, systematic and narrative reviews, methodological work on patient education, and case reports.

## Biomarkers and measures of treatment performance

Five contributions examine the measures used to characterise treatment performance and risk in prevalent dialysis populations. Zhang et al. measured plasma proenkephalin A 119–159 before and after a single haemodialysis session in one hundred patients, testing two properties reported in earlier populations, namely correlation with glomerular filtration rate and removal by extracorporeal therapy. Proenkephalin showed no association with estimated glomerular filtration rate in the whole cohort (r = −0.00, *p* = 0.99) or in the fifty-six patients with residual urine output (r = −0.15, *p* = 0.28), and concentrations were unchanged across the session (4.88 versus 4.80 ng/mL, *p* = 0.85) despite reductions in urea nitrogen from 12.59 to 3.68 mmol/L and creatinine from 458.70 to 165.85 *μ*mol/L. The authors conclude that the marker has limited value for assessing residual kidney function or dialysis adequacy in this population.

Zhong et al. conducted a single-arm, self-controlled study in twenty maintenance haemodialysis patients receiving one haemoperfusion session every two weeks in addition to high-flux dialysis, for twelve sessions. Indoxyl sulphate and p-cresyl sulphate decreased significantly within each combined session (*p* < 0.001), while pre-dialysis concentrations at the end of the programme did not differ from baseline. T lymphocyte subsets and the CD4+/CD8 + ratio increased, as did the proportion of cells with preserved mitochondrial membrane potential, and patients reported better sleep quality, less pruritus and improved quality of life (*p* < 0.01). Serum phosphorus and parathyroid hormone were also lower at the end of the treatment period. The authors state that these changes cannot be attributed mechanistically to sustained reduction of protein-bound uraemic toxins, and they list intermittent attenuation of peak concentrations, clearance of unmeasured solutes and non-specific effects among the possible explanations, presenting the findings as hypothesis-generating.

Li et al. assessed the C-reactive protein to albumin ratio against the Malnutrition-Inflammation Score in 231 prevalent haemodialysis patients, reporting an independent association with the score (OR 1.05, *p* < 0.001), an area under the receiver operating characteristic curve of 73.85% and an optimal cutoff of 2.158, together with a non-linear relationship between the two measures. A retrospective analysis of 275 patients followed for a median of fifty months (Cao et al.) compared machine learning algorithms with Cox regression for cardiovascular events and all-cause mortality. Gradient boosting performed best for cardiovascular events, with areas under the curve from 0.650 at one year to 0.755 at four years, and random forests best for mortality, from 0.862 to 0.931, with dialysis vintage, post-dialysis *β*2-microglobulin, collagen type I carboxy-terminal peptide, total bilirubin, lymphocyte count and roxadustat use among the contributing variables; the reported recall of 0.400 for the mortality model qualifies the discrimination figures and indicates that calibration and net clinical benefit require assessment before such models enter practice. In a mechanistic study of 62 insulin-treated patients with type 2 diabetes on dialysis and awaiting transplantation (D'Marco et al.), C-peptide concentrations correlated with epicardial adipose tissue volume, which in turn correlated with coronary artery calcification, while C-peptide and calcification showed no direct association, attributed by the authors in part to limited statistical power.

Across these five studies the quantity being measured and the outcome it is taken to represent come apart in different ways. Proenkephalin did not track residual kidney function and remained unchanged over a session in which urea and creatinine fell steeply, while the C-reactive protein to albumin ratio was validated against another index of malnutrition and inflammation, with no clinical endpoint involved. In the haemoperfusion study symptoms and immune measures improved while pre-dialysis toxin concentrations returned to baseline, so the clinical gain cannot be traced to the solutes the treatment targets. The machine learning comparison shows the same divergence inside a single analysis, since the mortality model combined areas under the curve above 0.86 with a recall of 0.400.

## Anaemia management

Three contributions address anaemia from complementary angles. Li et al. analysed 254 maintenance haemodialysis patients with 814 repeated measurements, linking linear mixed-effects trajectories of haemoglobin, log-ferritin and phosphorus to a Cox model within a joint modelling framework. Over a median follow-up of eighteen months, 62 patients died (24.4%), higher haemoglobin was independently associated with lower mortality (HR 0.97 per 10 g/L increase, 95% CI 0.95–1.00), log-ferritin showed a negative but non-significant association, phosphorus did not improve model fit, and the haemoglobin and log-ferritin model achieved time-dependent areas under the curve of approximately 0.70 to 0.75, with results stable in sensitivity analyses substituting transferrin saturation for ferritin.

Liu et al. examined determinants of haemoglobin target attainment in 4,906 patients across seventy dialysis centres in Wuhan between 2019 and 2023. Failure to reach 110 g/L was independently associated with female sex, tunnelled catheter use, dialysis frequency below three sessions weekly, pre-dialysis hypertension, absence of lanthanum carbonate use, higher serum phosphorus and higher C-reactive protein, while low-molecular-weight heparin use, employee medical insurance coverage, higher albumin, higher calcium and higher transferrin saturation were protective. Insurance coverage was included in the same model as iron and inflammatory indices, highlighting that aspects of anaemia control are influenced by the organisation of care itself.

Mi et al. compared two tapering strategies for roxadustat in 402 peritoneal dialysis patients from three centres in southern China who had reached target haemoglobin. Reducing administration frequency while holding the individual dose constant produced higher target attainment than dose reduction at three months (64.1% versus 40.4%), six months (55.6% versus 43.2%) and twelve months (49.2% versus 36.6%), lower haemoglobin variability by residual standard deviation (12.3 versus 15.5) and a lower adjusted hazard of target non-attainment (HR 0.64, 95% CI 0.50–0.82). The retrospective design and non-random allocation limit inference, and the question of how treatment should be withdrawn once a target is reached has received less formal study than the question of how it should be started. Across the three studies a haemoglobin value carries different information depending on the question asked of it: its trajectory over time predicts survival weakly but consistently, target attainment turns on conditions largely outside the haematological, and the tapering comparison indicates that the same value can be defended by more than one schedule.

## Mineral and bone disorder, vitamin D and diabetic kidney disease

Xu et al. review immune contributions to chronic kidney disease-mineral and bone disorder, covering cytokine-driven activation of the RANKL/RANK/osteoprotegerin axis together with NLRP3 inflammasome-dependent macrophage involvement in vascular calcification, and the effects of uraemic toxins on the balance between osteoblast and osteoclast activity. Sclerostin inhibition and FGF-23 modulation are discussed as candidate approaches, alongside sodium-glucose cotransporter 2 inhibition. They identify reliance on short-term surrogate endpoints such as parathyroid hormone and bone mineral density, together with population heterogeneity, as obstacles to clinical translation, a set of concerns that the recent KDIGO Controversies Conference on the same disorder examines in detail (2). The narrative review of vitamin D in chronic kidney disease (Wang et al.) covers non-dialysis, dialysis and transplant populations and reaches a practical position: nutritional vitamin D corrects deficiency and improves laboratory profiles, receptor activators suppress secondary hyperparathyroidism, and consistent effects on kidney failure, cardiovascular events, fractures and mortality remain undemonstrated, with heterogeneity of baseline status, formulation and dosing schedule among the likely explanations. On that basis the authors describe their own position as pragmatic, stopping short of any claim of disease modification: repletion of documented deficiency with nutritional vitamin D while avoiding high-dose bolus regimens, and use of active agents only where a clear indication exists, under monitoring of calcium, phosphate and parathyroid hormone; the framing is theirs, and it sits within the uncertainty their own synthesis describes. The review of diabetic kidney disease (Zhang et al.) addresses the population upstream of dialysis, setting the four established pharmacologic classes alongside ferroptosis, impaired autophagy, microbiota dysbiosis and epigenetic modification as emerging targets, and reporting evidence that simultaneous modulation of several pathogenic pathways confers greater cardiorenal protection than single-agent treatment. Its place in a Research Topic on dialysis-dependent disease follows from the composition of the dialysis population itself, since diabetic kidney disease supplies the largest single share of incident patients, and the pace at which progression to kidney failure is slowed upstream shapes the size and the comorbidity profile of the population that arrives on dialysis.

Luo et al. report a haemodialysis patient with calcific uraemic arteriolopathy, a condition carrying one-year mortality between 45% and 80%, in whom the disorder progressed after parathyroidectomy and in whom sodium thiosulfate produced substantial healing of cutaneous ulceration while tumoral calcinosis persisted. Cutaneous and metastatic calcification dissociated in the same patient, which bears on how response to treatment is judged in this setting.

## Symptom burden and infectious complications

Cao and Ma pooled fourteen randomised controlled trials comprising 1,232 participants on Niaoduqing granules for uraemic pruritus in Chinese haemodialysis populations. Pruritus scores fell in the treatment arm on both instruments assessed (visual analogue scale WMD −2.151, 95% CI −2.372 to −1.930; 5D-itch WMD −7.642, 95% CI −14.042 to −1.243), the response rate ratio was 1.249 (95% CI 1.169–1.335), adverse event rates were comparable with control across three trials, and parathyroid hormone, phosphorus, *β*2-microglobulin, high-sensitivity C-reactive protein, tumour necrosis factor-α and interleukin-6 differed significantly between groups. Sample sizes limit the strength of the conclusion, and this evidence has accumulated within a single health system, which shows how unevenly the literature on symptom control is distributed internationally.

A case report (Berrocal et al.) describes a 64-year-old man on long-term peritoneal dialysis with refractory peritonitis due to Streptococcus agalactiae, an uncommon pathogen in this setting, with peritoneal fluid showing 525 leukocytes/*μ*L and Gram-positive cocci. Intraperitoneal vancomycin produced no clinical improvement, and intravenous ampicillin-sulbactam at 1.5 g twelve-hourly, with oral fluconazole prophylaxis, achieved resolution after ten days, followed by catheter removal and transfer to intermittent haemodialysis. The choice of an intravenous regimen followed from the unavailability of the intraperitoneal formulations that guidelines recommend, a constraint documented across many countries in international surveys of kidney failure care (3). In this report, as in the meta-analysis before it, clinical decisions follow the treatments that are locally obtainable, a determinant of dialysis outcomes that rarely enters the trial literature as a measured variable.

## Education, self-management and service delivery

Lv et al. developed and validated an educational video for patients undergoing peritoneal dialysis catheter placement, using a five-stage methodological design with two Delphi rounds involving ten specialists and content validation by twenty incident patients. The final video covered eleven thematic areas from preoperative knowledge to home troubleshooting and rehabilitation guidance, with expert authority coefficients at or above 0.7, Kendall concordance above 0.3 after both rounds (*p* < 0.01), Cronbach *α* above 0.9 across dimensions and content validity indices above 0.9.

Zheng et al. followed 243 peritoneal dialysis patients over the first six months of treatment and identified three trajectories of fluid management behaviour, one increasing from a low level, one increasing from a medium level and one fluctuating between the two. Education level, diabetic nephropathy, peritonitis during the study period and hypoalbuminaemia distinguished the groups, physical activity scores rose within every trajectory (*p* < 0.001), and the ranking of groups by physical activity changed between early and later time points, indicating that self-management capability continues to develop after initiation.

Monshi et al. analysed Press Ganey survey responses from 5,472 dialysis patients attending Ministry of Health, Diaverum and DaVita facilities in Saudi Arabia during 2023. Overall satisfaction was 89.84 ± 14.25, highest in the personal experience domain and lowest in the dialysis domain itself, with private facilities scoring above governmental hospitals and female respondents above male respondents; sex and facility type were the significant predictors in regression analysis. These three studies move the object of assessment from the treatment to the person managing it at home and to the service that surrounds them, and in each case the measured quantity proves open to change.

## Outcomes outside the dialysis unit

Maman et al. analysed 1,957,284 total hip arthroplasties recorded in the Nationwide Inpatient Sample between 2016 and 2022, of which 2,730 involved dialysis-dependent patients. Unmatched in-hospital mortality was 1.3% against 0.03%, and after ten-to-one propensity score matching the dialysis-dependent group retained a mortality risk ratio of 10.1 (95% CI 6.3–16.2), with length of stay 62.5% longer and hospital charges 95.8% higher; major complications were also more frequent. Outcomes of elective surgery in dialysis-dependent patients fall between the responsibilities of nephrology and of the operating specialty, and the effect sizes reported here support shared perioperative pathways.

### Concluding remarks

Longitudinal modelling of repeated measurements replaces the single value in two of the anaemia studies and in the trajectory analysis of self-management behaviour, and this is the most visible of the features recurring across the collection. Negative and null findings are reported as such, in the assessment of proenkephalin and in the dissociation between toxin clearance and clinical improvement during haemoperfusion, while determinants outside the dialysis prescription, among them insurance coverage, facility ownership, education level and drug availability, enter the analyses as measured variables. Several important areas receive no coverage in this collection, including haemodiafiltration, incremental and home haemodialysis, conservative kidney management and treatment withdrawal, transplantation pathways, and health-economic analysis from lower-resource settings. Contributions came predominantly from China, with single studies from Saudi Arabia, Israel, and the United States.

Dialysis care is still governed by measures whose relationship to the outcomes that shape how patients live remains incompletely characterised, and the studies gathered here define parts of that gap with some precision.
